# Short term effect of intravenous treprostinil in term and preterm infants with pulmonary hypertension

**DOI:** 10.1186/s12887-023-04501-4

**Published:** 2024-01-29

**Authors:** Yoo-Jin Kim, Seung Han Shin, Ee-Kyung Kim, Han-Suk Kim

**Affiliations:** 1 Department of Pediatrics, Chung-buk National University Hospital, Cheongju-si, Republic of Korea; 2 Department of Pediatrics, Seoul National University College of Medicine, 101 Daehak-ro, Jongno-gu, Seoul, 03080 Republic of Korea; 3 Department of Pediatrics, Seoul National University Children’s Hospital, Seoul, Republic of Korea

**Keywords:** Pulmonary hypertension, Term infant, Preterm infant, Pulmonary vasodilator agent, Prostacyclin analogue

## Abstract

**Background:**

Pulmonary hypertension (PH) is a life-threatening condition in newborns. We aimed to assess the clinical and echocardiographic responses of term and preterm infants to treprostinil.

**Methods:**

This retrospective study included newborns diagnosed with PH and treated with treprostinil as additional therapy after inhaled nitric oxide administration in the neonatal intensive care unit of a tertiary center. Term and preterm infants were compared in terms of echocardiographic findings and clinical findings 4 weeks after treprostinil treatment.

**Results:**

During the study period, 11 term and 18 preterm infants were diagnosed with PH and received treprostinil. There were no differences in the echocardiographic findings of interventricular septal deviation, direction of shunt, and ratio of estimated pulmonary artery pressure over systolic blood pressure. Congenital diaphragmatic hernia was the most common condition occurring upon PH diagnosis among term infants, while severe bronchopulmonary dysplasia was the most common in preterm infants. Improvements in echocardiographic findings were more pronounced in term infants than in preterm infants (100% vs. 55.6%, P = 0.012). The inhaled nitric oxide dose was gradually tapered for term infants and was lower than that for preterm infants at 1, 2, and 3 weeks after treprostinil.

**Conclusion:**

Intravenous treprostinil could be an adjuvant therapy option for term and preterm infants with PH, especially for those who cannot receive oral medication. The efficacy and safety of treprostinil in this population with PH should be investigated further.

**Supplementary Information:**

The online version contains supplementary material available at 10.1186/s12887-023-04501-4.

## Background

Pulmonary hypertension (PH) is a life-threatening disease caused by various cardiopulmonary conditions in newborn infants; PH has high rates of morbidity and mortality [1]. In term infants, persistent pulmonary hypertension of the newborn (PPHN) is the most common cause of PH, and occurs in 1.9/1000 live-born term infants, with a mortality rate of 10% [2]. PH can also be caused by various perinatal conditions, such as lung hypoplasia, meconium aspiration syndrome, pneumonia, sepsis, transient tachypnea of the newborn, as well as congenital diseases such as congenital diaphragmatic hernia (CDH) and congenital heart disease (CHD) [1, 3]. In preterm infants, respiratory distress syndrome (RDS) can be accompanied by PPHN, and bronchopulmonary dysplasia (BPD) associated with PH has been characterized as a pulmonary vascular disease of the immature lung [4, 5]. The recently updated classification of PH categorized both CDH and BPD in the group of developmental lung disorders associated with PH [6].

A basic, yet important, strategy for the treatment of PH in newborn infants is the maintenance of body temperature, electrolytes, glucose, and intravascular volume [1]. Moreover, mechanical ventilation should be adequately supplied to improve oxygenation and achieve normal lung volumes. In severe cases, a pulmonary vasodilator agent is required to improve lung perfusion and subsequent systemic oxygenation. For example, inhaled nitric oxide (iNO), which selectively dilates pulmonary blood vessels, is the most widely used agent in newborn infants with PH [7]. Additional vasodilator therapies could be considered in a refractory case of PH, and several drugs indicated for the adult population, including sildenafil and bosentan, have also been used in newborn infants [8].

Treprostinil is a synthetic analog of prostacyclin that stimulates adenylyl cyclase in vascular smooth muscle cells, leading to increased levels of intracellular cyclic AMP and subsequent vasodilation in the systemic and pulmonary circulatory systems [9]. The drug can be administered parenterally and, hence, could be useful as an add-on therapy for intractable cases involving newborn infants who cannot tolerate enteral feeding. Although several studies have shown the efficacy of treprostinil in term infants with CDH or PPHN [10–13], reports on its use in preterm infants remain scarce [14].

This study aimed to assess the clinical and echocardiographic responses of newborn infants to treprostinil and investigate whether there are differences in the effects of treprostinil between term and preterm infants.

## Methods

This retrospective study included newborn infants who were admitted to the neonatal intensive care unit of Seoul National University Children’s Hospital between January 2015 and October 2020. Infants who were diagnosed with PH and treated with intravenous treprostinil as an add-on therapy of iNO were included, while infants who died within 72 h of treprostinil administration and did not reach the therapeutic dose of 20 ng/kg/min or who were treated with extracorporeal membrane oxygenation were excluded. The study population was categorized as term (≥ 37 weeks) or preterm (< 37 weeks) infants, according to the gestational age.

Treprostinil was used as a rescue therapy after iNO treatment when there was no clinical or echocardiographic improvement with the invasive respiratory support of FiO_2_ > 0.5, and when enteral medication was limited or had already been administered to the patients. It was administered through continuous intravenous infusion and injected through a central venous catheter, with the initial infusion rate at 2 ng/(kg min) and increasing incrementally by 2 ng/(kg min) every 8–12 h, depending on the response to the drug. Tapering of drugs was attempted based on the improvement indicated by the clinical and echocardiographic findings.

PH was diagnosed using transthoracic echocardiography based on the following findings: right-to-left or bidirectional shunt, velocity of tricuspid regurgitation ≥ 3 m/s, or left-deviated or flat configuration of the interventricular septum [15] (Supplementary Table S1). The pressure gradient between the right atrium and right ventricle was calculated using a modified Bernoulli equation, and pulmonary artery pressure (PAP) was estimated by adding 5 mm Hg to the pressure gradient [16]. PAP divided by systolic blood pressure (PAP/sBP) was compared before and after treprostinil treatment between the two groups. Ejection fraction (EF) was calculated by measuring left ventricle end-diastolic volume and left ventricle end-systolic volume and using Simpson’s method. Ejection fraction (EF) was calculated by measuring end-systolic and end-diastolic LV internal diameters in M-mode. Echocardiographic improvement was defined as an improvement in septal deviation, shunt direction, or reduction in PAP/sBP > 30%, and those who experienced the improvement during the 4 weeks of the study period were defined as responders (Supplementary Table S1). Medical records were also reviewed, including the use of treprostinil, associated conditions of PH, mode of ventilator support, use of inotropic agents for hypotension, and the use of other PH drugs. The respiratory severity score (RSS) was calculated as mean airway pressure (MAP) × FiO_2_ and was compared between groups [17]. Echocardiographic findings and clinical findings before treprostinil treatment and 4 weeks after treprostinil treatment were compared between term and preterm infants. The study was approved by the Institutional Review Board of Seoul National University Hospital (IRB No. 2105-081-1218). The requirement of obtaining informed consent was waived by the Institutional Review Board of Seoul National University Hospital due to the retrospective nature of the study.

For statistical analysis, the Wilcoxon rank sum test was used to compare continuous variables, and Fisher’s exact test was used for categorical variables. Data are expressed as percentage values or median (interquartile range). Statistical significance was set at P < 0.05. Data analysis was performed using STATA version 12.0 for Windows (Stata Corp., College Station, Texas, United States of America).

## Results

**Fig. 1:**
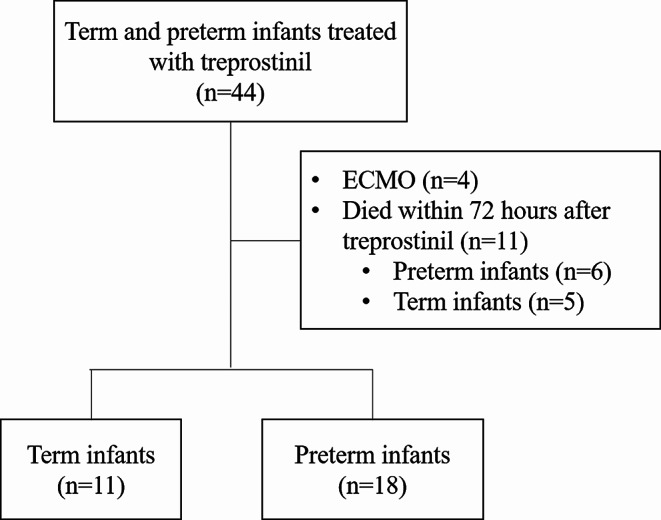
Flow chart of the study population. ECMO, extracorporeal membrane oxygenation

Among 44 newborn infants who received treprostinil due to pulmonary hypertension during the study period, 11 term and 18 preterm infants were finally included in this study (Fig. 1).

**Table 1:** Demographic and baseline characteristics of the study population

	Term infants (n = 11)	Preterm infants (n = 18)	p value
GA (weeks)	39.9 [39.4–40.3]	30.8 [24.9–33.4]	< 0.001
Birthweight (grams)	3180 [3050–3690]	810 [700–2050]	< 0.001
SGA	1 (9.1)	3 (16.7)	1.000
Female	6 (54.6)	6 (33.3)	0.438
PH diagnosis (postnatal days)	1 (1–1)	6 (2–19)	0.001
**Associated conditions**			
PPHN	3 (27.3)	4 (22.2)	1.000
Congenital DH	8 (72.7)	3 (16.7)	0.005
Severe BPD	0 (0)	10 (62.5)	0.001
**At the time of treprostinil administration**			
Start day (postnatal day)	4 [2–13]	18.5 [7–54]	0.012
Weight (gm)	3180 [3080–3510]	1190 [530–2620]	0.021
*Respiratory support*			
Mode of ventilation			0.202
Conventional vent	1 (9.1)	6 (33.3)	
HFOV	10 (90.9)	12 (66.7)	
FiO_2_	0.8 [0.6–0.8]	0.8 [0.7–0.95]	0.224
MAP (cmH_2_O)	14 [11–15]	13.5 [12–16]	0.957
RSS	10.4 [7.2–12]	11.1 [8.4–13.6]	0.499
iNO (ppm)	50 [40–60]	55 [40–80]	0.843
PH drug other than iNO	3 (27.3)	11 (61.1)	0.128
*EchoCG*			
PAP/sBP	1 [0.78–1.38]	0.79 [0.62–1.03]	0.134
R to L or bidirectional shunt	4 (36.4)	6 (33.3)	1.000
Interventricular septal deviation	10 (90.9)	15 (83.3)	1.000
Ejection fraction (%)	70 [56.3–82.7]	72.4 [58.4–79.5]	0.792

Values are expressed as n (%) or median [interquartile range]. GA, gestational age; SGA, small for gestational age; PH, pulmonary hypertension; CDH, congenital diaphragmatic hernia; PPHN, persistent pulmonary hypertension; BPD, bronchopulmonary dysplasia; HFOV, high frequency oscillatory ventilation; MAP, mean airway pressure; RSS, respiratory severity score; iNO, inhaled nitric oxide; EchoCG, echocardiography; PAP, pulmonary arterial pressure; sBP, systolic blood pressure

CDH was the most common condition at diagnosis of PH in term infants, and severe BPD was the most common in preterm infants (Table 1). Treprostinil treatment was started earlier in term infants than in preterm infants (4.0 vs. 18.5 days, P = 0.012). When initiating treprostinil administration, high-frequency oscillatory ventilation (HFOV) was used in 90.9% and 66.7% of term and preterm infants, respectively (P = 0.202). No significant differences in FiO_2_ (0.8 vs. 0.8, P = 0.224) or mean airway pressure (MAP) (14 vs. 13.5 mmHg, P = 0.957) were observed between term and preterm infants. Similarly, RSS values were comparable between the two groups (10.4 vs. 11.1, P = 0.499). Echocardiographic evaluation showed that PAP/sBP was comparable between the two groups (1.0 vs. 0.8, P = 0.134). The rate of right-to-left and/or bidirectional shunt via patent ductus arteriosus or an atrial septal defect, as well as deviation of the interventricular septum, were also comparable between the two groups.

**Table 2:** Respiratory and echocardiographic findings at the initiation of treprostinil

	Term infants (n = 11)	Preterm infants (n = 18)	p value
Maximum dose of treprostinil (ng/(min kg))	40 [40–52]	40 [26–50]	0.453
Duration of treprostinil (days)	24 [22–43]	24 [16–32]	0.921
**Clinical courses during 4 weeks**			
Mode of ventilation			0.039
Non-invasive ventilation	7 (63.6)	2 (14.3)	
Conventional vent	3 (27.3)	6 (42.9)	
HFOV	1 (9.1)	6 (42.9)	
Adding inotropics for hypotension	1 (9.1)	6 (46.2)	0.078
Increase in inotropics for hypotension	5 (45.5)	12 (66.7)	0.438
Withholding treprostinil due to hypotension	0 (0)	1 (5.6)	1.000
Adding another PH drug	5 (45.5)	3 (21.4)	0.389
Death	0 (0)	7 (38.9)	0.026
**Post-treprostinil EchoCG**			
PAP/sBP	0.6 [0.4–0.9]	0.5 [0.4–0.6]	0.274
R to L or bidirectional shunt	0 (0)	1 (6.7)	1.000
Interventricular septal deviation	4 (36.4)	4 (26.7)	0.683
Ejection fraction (%)	71.9 [68.7–77.6]	74.1 [63.9–79.4]	0.860
Any improvement in EchoCG	11 (100)	10 (55.6)	0.012

Values are expressed as n (%) or median [interquartile range]. HFOV, High-frequency oscillatory ventilation; PH, pulmonary arterial hypertension; EchoCG, echocardiography; PAP, pulmonary arterial pressure; sBP, systolic blood pressure

**Fig. 2:**
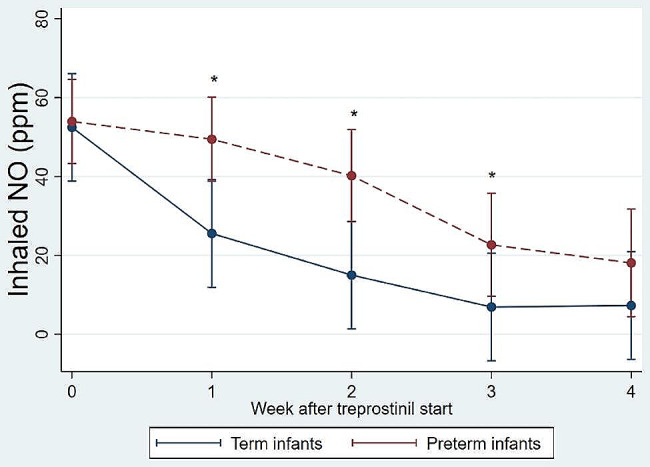
Changes in the inhaled NO dose after treprostinil therapy. NO, nitric oxide. * indicates a p-value < 0.05

For both groups, the maximum infusion rate of treprostinil was 40 ng/(min kg), and the duration of treprostinil infusion was 24 days in all cases (Table 2). Follow-up echocardiographic evaluation 4 weeks post-treprostinil treatment showed no differences in PAP/sBP, shunt direction, septal deviation, or ejection fraction between the two groups. However, all term infants showed improvement in echocardiography, while only 55.6% of preterm infants showed improvement in echocardiography (p = 0.012). The post-hoc power calculation for the comparison of improvement in echocardiography was 82.4%. More term infants could be weaned off non-invasive ventilation than preterm infants, while a higher proportion of infants were supported by HFOV in the preterm group compared to that in the term group (9.1% vs. 42.9%, P = 0.039). The addition of inotropic agents such as dopamine and dobutamine was required to increase blood pressures more often in preterm infants, although this difference was not statistically significant (9.1% vs. 46.2%, P = 0.078). Increase in dosage of any inotropic agent for hypotension occurred in 45.5% of term infants and 66.7% of preterm infants (p = 0.438). There was only one preterm infant in whom treprostinil was stopped due to refractory hypotension. All term infants survived during the 4 weeks of treprostinil treatment, while seven preterm infants (38.9%) died during the first 4 weeks of treatment. The causes of death in preterm infants were refractory pulmonary hypertension in four cases, severe respiratory failure in two cases, and sepsis in one case. iNO dose was gradually decreased in term infants and was lower than that administered to preterm infants at 1, 2, and 3 weeks after treprostinil administration (Fig. 2).

**Table 3:** Clinical courses before discharge

	Term (n = 11)	Preterm (n = 18)	p value
Treprostinil use more than 4 weeks	3 (27.3)	4 (22.2)	1.000
Death before discharge^a^	2 (18.2)	11 (61.1)	0.052
Weaning from treprostinil before death or discharge	11 (100)	9 (50)	0.005
Requiring other PH medication after treprostinil than iNO^b^	6 (54.6)	7 (77.8)	0.374
Duration of other PH medication (days)^b^	73.5 [68–94]	169 [40–938]	0.412

Values are expressed as n (%) or median [interquartile range]. PH, pulmonary hypertension; iNO, inhaled nitric oxide

^a^Including death during 4 weeks of study period

^b^Among infants who could be weaned from treprostinil before death

After the 4-week study period, 3 term and 4 preterm infants were still treated with treprostinil (Table 3). Overall, two term (18.2%) and 11 preterm (61.1%) infants died before discharge (p = 0.052). However, all the term infants could be weaned from treprostinil before death or discharge, whereas only 50% of preterm infants could be weaned from treprostinil (p = 0.005). Among infants who were weaned from treprostinil before death, 6 (54.6%) term and 7 (77.8%) preterm infants required medications for pulmonary hypertension other than inhaled nitric oxide (iNO) at the time of treprostinil withdrawal (p = 0.374). Sildenafil was used in 6 term and 10 preterm infants. Bosentan or ambrisentan was used in 3 term and 6 preterm infants. Inhaled iloprost was used in 2 preterm infants. The number of patients who required two or more medications at the timing of treprostinil discontinuation was 3 and 6 among the term and preterm infants, respectively. Median duration of other medication was 73.5 days in term infants and 169 days in preterm infants (p = 0.412) from initiation of treprostinil.

Among the 18 preterm infants, 11 showed no improvement during 4 weeks of study period in echocardiographic findings (Table 4). Of the preterm infants, two with CDH, four with severe BPD, four with PPHN including one with Potter sequence, and one with Down syndrome did not respond to treprostinil. Meanwhile, one preterm infant with CDH and six with severe BPD showed improvement in echocardiographic findings.

Subgroup analysis was conducted in infants with CDH. There were 8 term with CDH and 3 preterm infants with CDH (Supplementary Table S2). In the demographic findings, there were no differences between the two groups except gestational age, birthweight, and weight at initiation of treprostinil. During the study period, more preterm infants required additional inotropics for hypotension (12.5% vs. 100%, p = 0.024). Mortality was more prevalent in preterm infants with borderline significance (0% vs. 66.7%, p = 0.055). All term infants showed improvement in echocardiographic findings, while two out of three preterm infants showed improvement. (p = 0.152).

**Table 4:** Characteristics of responders and non-responders to treprostinil in echocardiography among preterm infants

	GA (weeks)	Birthweight (gm)	Sex	Associated conditions	Treprostinil (PND)	Conditions at treprostinil start					Outcomes
						Weight (g)	Mode of ventilator	RSS	Inhaled NO (ppm)	At 4 weeks	At discharge
Non-responder	34.7	2,540	Male	CDH	1	2,540	HFOV	9.8	70	Died	
	34	2,050	Male	CDH	7	2,430	HFOV	8	80	Died	
	31.1	1,210	Male	Down syndrome	73	2,620	HFOV	11	80	Died	
	23.6	660	Male	PPHN	11	920	HFOV	12.4	40	Died	
	27.4	1,140	Male	PPHN	4	1,440	HFOV	8.4	50	Survived	Survived
	34.7	2,600	Female	PPHN	14	2,650	HFOV	13.6	80	Died	
	33.1	2,010	Male	PPHN, Potter sequence	1	2,010	HFOV	11.2	60	Died	
	28.4	390	Female	Severe BPD	314	7,350	HFOV	8.4	20	Survived	Survived
	26.9	830	Female	Severe BPD	298	4,920	Conventional	13.6	11	Survived	Survived
	24.9	770	Female	Severe BPD	39	1,460	HFOV	16	40	Survived	Survived
	24.4	710	Male	Severe BPD	18	700	Conventional	12.4	80	Died	
Responder	35.7	2,340	Male	CDH	10	2,900	HFOV	8.4	40	Survived	Died
	30.6	600	Male	PPHN, Severe BPD	4	530	HFOV	6.2	40	Survived	Survived
	24.9	700	Male	Severe BPD	19	850	Conventional	6	30	Survived	Died
	24.4	690	Female	Severe BPD	32	890	HFOV	6.6	60	Survived	Survived
	33.4	2,490	Female	Severe BPD	121	5,110	Conventional	12.9	60	Survived	Survived
	30.9	740	Male	Severe BPD,	45	1,340	Conventional	18	40	Survived	Died
	31.9	790	Male	EA, severe BPD, chylothorax	54	1,750	Conventional	16.1	80	Survived	Died

GA, gestational age; PND, postnatal days; CDH, congenital diaphragmatic hernia; PPHN, persistent pulmonary hypertension; BPD, bronchopulmonary dysplasia; HFOV, high frequency oscillatory ventilation; EA, esophageal atresia

## Discussion

This study evaluated short-term responses to treprostinil in newborn infants and showed that the clinical and echocardiographic responses following intravenous treprostinil administration for 4 weeks differed between term and preterm infants. Although pre-treatment conditions such as RSS, iNO requirement, and echocardiographic findings were comparable between the two groups at the start of treprostinil administration, improvement in any echocardiographic findings were more commonly found in term infants with lower iNO doses at 1, 2, and 3 weeks. Preterm infants tended to receive additional inotropic agents to control decreased blood pressure during treprostinil administration; however, the difference was not statistically significant. Experiences involving the use of treprostinil in the pediatric population have been reported. A retrospective cohort study of children with PH treated with treprostinil showed early and sustained improvement in RV function [18]. In another retrospective study including patients under 12 months of age, treprostinil for the treatment of neonatal diseases was well tolerated [19].

The effects of treprostinil might be influenced by the underlying or accompanying conditions of PH among term and preterm infants. In our study, CDH was often accompanied by PH treated with treprostinil in term infants, whereas BPD was the most common condition in preterm infants. Carpentier et al. reported that treprostinil was used as a rescue therapy in 14 infants diagnosed with CDH, and clinical and echocardiographic improvements were achieved with treprostinil treatment in 12 infants [11]. In another study, 17 infants with CDH were treated with treprostinil as rescue therapy, and a decrease in PH severity was observed 1 month post treatment with 11 of the 17 infants surviving [12]. The survival of term infants with CDH treated with treprostinil in our study was comparable with the results of previous studies, regarding improvement in echocardiographic findings. However, two preterm infants with CDH who received treprostinil died within 4 weeks after treatment, and another infant who showed improvement in echocardiography eventually died before discharge. Poor response to treprostinil in preterm infants with CDH might be attributable to immature cardiopulmonary circulation and lung conditions, which could worsen the condition of underdevelopment of the lung in CDH [20].

There have been few reports on the usage of treprostinil in preterm infants owing to the limited number of patients. A case report of two preterm infants diagnosed with sepsis-associated PH that was intractable to iNO demonstrated that clinical improvement was achieved within 12 h after treprostinil was started [21]. In a retrospective study, five preterm infants diagnosed with BPD with severe PH were treated with subcutaneous treprostinil and showed improved echocardiographic findings in right ventricular function, decreased PH severity, and decreased requirement for respiratory support [14]. In that study, treprostinil was started at 20–152 weeks after birth, with a maximum dose of 20–50 ng/(kg min). In the present study, ten preterm infants with BPD-associated PH were treated with treprostinil. Among them, six infants showed improvement over 4 weeks, while the other four infants were classified as non-responders. Weight at birth or treprostinil might not be contributing factors for the response to the drug, since these factors were comparable between non-responders and responders, and three infants weighing < 1,500 gm at the time of treatment were also included in the responder group. Rather, the pathobiology of the PH might be important in determining the effect of treprostinil, as two infants with severe BPD in the non-responder group still required invasive ventilation beyond 10 months of age, implicating a profound developmental problem of the lung. Moreover, those with underlying conditions such as Down syndrome and Potter sequence showed no improvement on echocardiography.

Drawing a conclusion on the effectiveness of the drug based on these results should be approached with caution, as different responses of PH to treprostinil in term and preterm infants might be attributed to other factors, such as the timing of treprostinil initiation and the use of treprostinil as a second- or higher-line choice in the management of PH. In this study, treprostinil was administered to term infants in the early postnatal period, during which, delayed transition from fetal circulation could have contributed to PH, although this can be resolved over time [1]. Furthermore, as the surgical condition of CDH was more common in term infants, enteral feeding and administration of oral medication such as sildenafil and bosentan were limited. Therefore, treprostinil was used as a next-line therapy after iNO in term infants, and as a third-or fourth-line option after using oral medications for intractable PH in preterm infants. In the study population, other PH drugs were already used in 61.1% of preterm infants and 27.3% of term infants at the start of treatment. Furthermore, the nature of the underlying disease may serve as a contributing factor in the response to treprostinil. For instance, diseases such as PPHN in term infants may exhibit a transitional nature of pathophysiology [2]. In contrast, BPD is characterized by a developmental nature, necessitating a longer duration for improvement [4].

Apart from being a retrospective study with a small sample size, this study has several limitations. The variety of underlying or accompanying conditions posed a challenge in interpreting differences in the efficacy of the drug between term and preterm infants. In this study, 6 preterm and 5 term infants who died within 72 h after infusion were excluded, as they were in deteriorating conditions that did not respond to any medical interventions. As the infusion rate was increased gradually every 8 h, they died (mostly within 48 h after infusion) before the infusion rate reached 20 ng/(min kg). Moreover, the study did not include pharmacokinetic data, which are critical factors that assist in explaining and understanding the different clinical responses between groups. Additionally, long-term pulmonary and neurodevelopmental outcomes were not explored in this study.

## Conclusion

Intravenous treprostinil is a potential option for rescue therapy in term and preterm infants with PH, especially in situations where oral medication use is limited owing to underlying conditions or the unavailability of other parenteral medications. However, further investigation into the efficacy of this drug in term and preterm infants is warranted.

### Electronic supplementary material

Below is the link to the electronic supplementary material.

**Supplementary Material 1:** Diagnosis of pulmonary hypertension

## Data Availability

The data that support the findings of the current study are available from the corresponding author on reasonable request.
