# Supplementary material for: Short term effect of intravenous treprostinil in term and preterm infants with pulmonary hypertension
Source: BMC Pediatr. 2024 Jan 29;24:83. doi: 10.1186/s12887-023-04501-4 (PMC10823739; doi:10.1186/s12887-023-04501-4)
Supplement: Supplementary file 1 — Supplementary Material 1: Diagnosis of pulmonary hypertension [file 12887_2023_4501_MOESM1_ESM.docx]

**Supplementary material**

**Short term effect of intravenous treprostinil in term and preterm infants with pulmonary hypertension**

Yoo-Jin Kim^1^, Seung Han Shin^2,3^, Ee-Kyung Kim^2,3^, Han-Suk Kim^2,3^

^1^ Department of Pediatrics, Chung-buk National University Hospital, Republic of Korea

^2^ Department of Pediatrics, Seoul National University College of Medicine, Republic of Korea

^3^ Department of Pediatrics, Seoul National University Children’s Hospital, Republic of Korea

Corresponding Author:

Seung Han Shin, MD

Department of Paediatrics, Seoul National University College of Medicine

Seoul National University Children’s Hospital, 101 Daehak-ro, Jongno-gu, Seoul 03080, South Korea

Tel: +82-2-2072-7230

Fax: +82-2-747-5130

E-mail: revival421@snu.ac.kr

**Table S1. Diagnostic criteria for PH and definition of improvement in PH on echocardiography**

| Diagnosis of PH (any of the following) |
| --- |
| - Right-to-left or bidirectional shunt |
| - Velocity of tricuspid regurgitation ≥3 m/s |
| - Left-deviated or flat configuration of the interventricular septum |
| Improvement in PH (any of the following) |
| - Improvement in septal deviation |
| - Improvement in shunt direction |
| - Reduction in PAP/sBP >30% |

PH, pulmonary hypertension; PAP, pulmonary arterial pressure; sBP, systolic blood pressure

| **Table S2. Characteristics and treprostinil related data of infants with CDH** | | | |
| --- | --- | --- | --- |
|  | Term (n=8) | Preterm (n=3) | p value |
| GA (week) | 39.7 [39.2-40.2] | 34.7 [34-35.7] | 0.012 |
| Birthweight (gram) | 3210 [3115-3600] | 2340 [2050-2540] | 0.012 |
| Female | 62.5 (54.6) | 0 (33.3) | 0.182 |
| PH diagnosis (postnatal days) | 1 [1-1] | 1 [1-3] | 0.436 |
| At the time of treprostinil administration |  |  |  |
| Start day (postnatal day) | 2.5 [1.5-5.5] | 7 [1-10] | 0.667 |
| Weight (gm) | 3355 [3165-3600] | 2540 [2430-2900] | 0.012 |
| Respiratory support (all HFOV) |  |  |  |
| FiO_2_ | 0.8 [0.58-0.8] | 0.75 [0.6-0.8] | 0.788 |
| MAP | 13.5 [12-14.5] | 13 [10-14] | 0.582 |
| RSS | 9.7 [7.98-11.55] | 8.4 [8-9.75] | 0.497 |
| iNO (ppm) | 50 [42.5-60] | 70 [40-80] | 0.327 |
| EchoCG |  |  |  |
| PAP/sBP | 1.08 [0.86-1.32] | 0.92 [0.6-1.16] | 0.497 |
| R to L or bidirectional shunt | 2 (25) | 2 (66.7) | 0.491 |
| Interventricular septal deviation | 2 (25) | 2 (66.7) | 0.491 |
| Ejection fraction (%) | 72.05 [49.3-83.47] | 54 [53.81-74] | 0.497 |
| Maximum dose of treprostinil (ng/(min∙kg)) | 40 [40-46] | 40 [26-50] | 0.933 |
| Duration of treprostinil (days) | 25 [22.5-35] | 20 [16-32] | 0.497 |
| Adding inotropics for hypotension | 1 (12.5) | 3 (100) | 0.024 |
| Adding other PAH drug | 4 (50) | 2 (66.7) | 1.000 |
| Death | 0 (0) | 2 (66.7) | 0.055 |
| Post-treprostinil EchoCG |  |  |  |
| PAP/sBP | 0.63 [0.52-0.67] | 0.52 [0.49-0.78] | 0.786 |
| R to L or bidirectional shunt | 0 (0) | 1 (33.3) | 0.273 |
| Interventricular septal deviation | 2 (25) | 1 (33.3) | 1.000 |
| Ejection fraction (%) | 71.9 [71.8-76] | 80.7 [77-84.2] | 0.143 |
| Any improvement in EchoCG | 8 (100) | 2 (66.7) | 0.152 |

Values are expressed as n (%) or median [interquartile range]. CDH, congenital diaphragmatic hernia; GA, gestational age; PH, pulmonary hypertension; HFOV, high frequency oscillatory ventilation; MAP, mean airway pressure; RSS, respiratory severity score; iNO, ihaled nitric oxide; EchoCG, echocardiography; PAP, pulmonary arterial pressure; sBP, systolic blood pressure; TAPSE, tricuspid annular plane systolic excursion.
